# Effect of Different Purification Techniques on the Characteristics of Heteropolysaccharide-Protein Biopolymer from Durian (*Durio zibethinus*) Seed

**DOI:** 10.3390/molecules170910875

**Published:** 2012-09-10

**Authors:** Bahareh Tabatabaee Amid, Hamed Mirhosseini

**Affiliations:** Department of Food Technology, Faculty of Food Science and Technology, University Putra Malaysia, 43400 UPM Serdang, Selangor, Malaysia; Email: bahareh.ta@gmail.com

**Keywords:** biopolymer, gum purification, heteropolysaccharide, solubility, durian seed

## Abstract

Natural biopolymers from plant sources contain many impurities (e.g., fat, protein, fiber, natural pigment and endogenous enzymes), therefore, an efficient purification process is recommended to minimize these impurities and consequently improve the functional properties of the biopolymer. The main objective of the present study was to investigate the effect of different purification techniques on the yield, protein content, solubility, water- and oil-holding capacity of a heteropolysaccharide-protein biopolymer obtained from durian seed. Four different purification methods using different chemicals and solvents (*i.e.*, A (isopropanol and ethanol), B (isopropanol and acetone), C (saturated barium hydroxide), and D (Fehling solution)] to liberate the purified biopolymer from its crude form were compared. In most cases, the purification process significantly (*p* < 0.05) improved the physicochemical properties of heteropolysaccharide-protein biopolymer from durian fruit seed. The present work showed that the precipitation using isopropanol and acetone (Method B) resulted in the highest purification yield among all the tested purification techniques. The precipitation using saturated barium hydroxide (Method C) led to induce the highest solubility and relatively high capacity of water absorption. The current study reveals that the precipitation using Fehling solution (Method D) most efficiently eliminates the protein fraction, thus providing more pure biopolymer suitable for biological applications.

## 1. Introduction

Natural gums are usually complex polymers, which can be found in various sources such as the endosperm of plant seeds (guar gum), plant exudates (e.g., tragacanth), and tree or shrub exudates (e.g., gum Arabic and tragacanth). Natural gums are also originated from the other sources such as seaweed (e.g., agar), bacteria (e.g., xanthan gum) and animal sources (e.g., hyaluronan, chitin and chondroitin sulphate) [1,2]. In recent years, the demand for natural hydrocolloids from plant sources has increased considerably because they are the most notable food ingredient in liquid and semisolid foods [2]. They are used for widespread industrial applications such as dietary fiber, texture modifiers, gelling agents, thickeners, stabilizers and emulsifiers, coating agents, binders and drug release modifiers [1,2,3,4]. Most natural gums are safe for oral consumption and are preferred over analogous synthetic gums due to their non-toxicity, low cost and availability [4,5]. However, there are some technical restrictions for the application of natural plant gums in food, cosmetic and pharmaceutical products. The selection of an appropriate gum is based on certain criteria such as solution clarity, solubility at various temperatures, suspension ability, natural or synthetic, and ability to stabilize proteins at a low pH, acid stability, or relative cost per pound [4]. Many natural gums are used in high concentrations, in order to exhibit their actual functions (e.g., as drug release modifiers). Therefore, the application of further physical and/or chemical treatments to improve the physicochemical and functional properties of natural gum from plant sources is recommended. 

Chemical purification minimizes these drawbacks at the same time that it provides better function for specific purposes (e.g., drug delivery) [4]. It can also improve the unacceptable flavor and color of the crude polysaccharide gums by the removal of impurities (e.g., fat, protein, fiber, natural pigment and endogenous enzymes) during the purification process [6]. Finally, the purified gums give clearer and more stable solutions, due to the elimination of impurities [6]. There are several suggested techniques for the purification of crude plant gums. Precipitation using alcohols (*i.e.*, ethanol and methanol) was reported by previous researchers [7,8]. Previous researchers also reported other purification techniques for various plant gums with copper or barium complexes [9,10] and borate buffer [11]. In addition, the precipitation with isopropanol is one of the commonly used methods for the purification of crude gums on the industrial scale [12,13]. Therefore, it is necessary to screen the suitable purification technique in order to improve the functional characteristics of crude durian seed gum. The main goal of the current study was to compare the efficiency of different purification techniques by assessing the purification yield, protein content, solubility, water and oil holding capacity (WHC and OHC) of biopolymer from durian (*Durio zibethinus*) seed. To the best of our knowledge, there are no similar studies investigating the effect of different chemical purification methods on the physicochemical properties of crude durian seed gum.

## 2. Results and Discussion

### 2.1. Purification Yield

**Figure 1:**
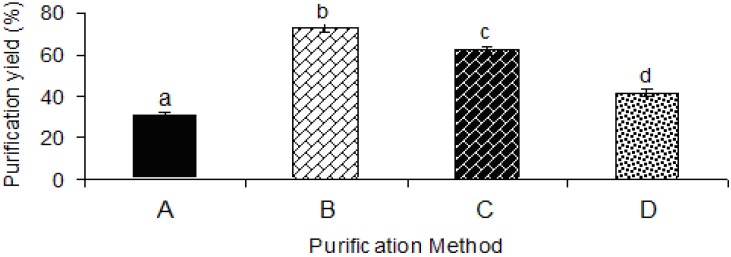
Effect of different purification methods on the purification yield. A, isopropanol and ethanol; B, isopropanol and acetone; C, saturated barium hydroxide; D, Fehling solution.

In the current study, the chemical purification along with further centrifugation provided a more efficient process for the elimination of impurities than either the simple chemical purification or centrifugation alone. The efficiency of the purification method was mainly dependent upon the purification procedure and composition of the utilized solvents and reagents. The results indicated that the purification yield was significantly (*p* < 0.05) influenced by the purification process (Figure 1). In fact, the yield of purification varied depending on the basic composition of reagents utilized for purification treatment. On the other hand, the yield also depends on the time and temperature required for each purification process. The purification methods provided significantly (*p* < 0.05) different purification yields in the following order: Method B (isopropanol and acetone) > Method C (saturated barium hydroxide) > Method D (Fehling solution) > Method A (isopropanol and ethanol). The purification yields ranged from 30.9% to 72.4%, depending on the purification method (Figure 1). This value was higher than the purification yield reported for fenugreek gum (22%) [14], locust bean gum (26.66%–33.16%) [6] and almost similar to that reported for guar gum (22%–78%) [15]. Cunha *et al.* revealed that the purification yield depended on the method applied [15]. 

Among all the purification techniques, Method B and Method A gave the highest (72.4%) and lowest (31.0%) purification yield, respectively (Figure 1). Since, isopropanol was utilized in both purification methods (A and B); therefore, the difference could be explained by a greater effectiveness of acetone rather than ethanol. The results indicated that the purification methods C and D showed relatively high purification yields (62.4% and 41.6%), respectively (Figure 1).

The low purification yield obtained with Method A could probably be due to the short time used for dissolving the crude sample. Previous researchers [15] have also reported that the low purification yield might be due to the short contact time. The low purification yield could also be due to the mixture of isopropanol and water. In heteropolysaccharide-protein biopolymer (*i.e.*, durian seed gum), the interaction between the sample matrix and solvents depends on both polysaccharide and protein fractions present in the molecular structure of the biopolymer. For instance, the mixture of isopropanol and water causes a high dielectric constant, where the protein fraction is more soluble in isopropanol rather than water; while the polysaccharide fraction is more soluble in water rather than isopropanol. If both fractions are more soluble in the same solvent, the purification yield will be more desirable than solubility in different solvents. The present study revealed that the purification using isopropanol and acetone (Method B) and saturated barium hydroxide (Method C) provided the highest purification yield. This could be probably interpreted by the recovery of almost all the polysaccharide precipitated with isopropanol and acetone.

The purification process reduces the content of main impurities (such as trace elements, tannin, natural pigments and protein) present in the chemical structure of the crude gum. Thus, the efficiency of chemical purification process using different organic solvents mainly depends on several factors such as the sample matrix as well as type and content of impurities (e.g., fat, fiber, protein, enzymes and minerals). On the other hand, the effectiveness of the purification method is influenced by the processing conditions such as the polarity and dielectric constant of the solvents utilized and reagents. The current study revealed that acetone was a much better solvent than ethanol and other reagents for achieving maximum purification yield. However, the yield does not reflect the feasibility of the purification method. In fact, the screening of the most efficient purification method is based on not only the yield, but also the physicochemical and functional properties of the purified gum. Although a purification process may provide high processing yield, but it might not induce the desirable functional properties too, so it is necessary to investigate the effects of the purification process on the physico-chemical and functional properties of the resulting gum.

### 2.2. Protein Content

**Figure 2:**
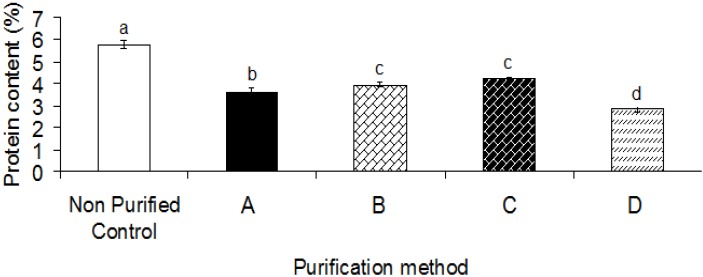
Effect of different purification methods on the protein content of heteropolysaccharide-protein biopolymer from durian seed. A, isopropanol and ethanol; B, isopropanol and acetone; C, saturated barium hydroxide; D, Fehling solution.

Protein analysis indicated that all purification processes significantly (*p* < 0.05) decreased the protein content (2.65%–4.10%) as compared to the control sample (or crude gum) (5.8%, Figure 2). The purified gum provided significantly (*p* < 0.05) different protein contents by the following order: crude gum > purified gum C ≥ purified gum B > purified gum A > purified gum D. The reduction of protein content is considered as a key biological aspect of the gum. 

As reported by Cunha *et al.* [15], the purified gum containing a low amount of the protein fraction is more pure and appropriate than the crude gum with a high protein content for biological purposes. Therefore, the purification method resulting in the most significant reduction of the protein fraction is recommended, if the biological aspect of the gum is targeted. On the other hand, the “protein fraction” can be responsible for the surface activity and this hydrophobic nature is an obligatory requirement for the gums to adsorb on interfaces. As stated by Dickinson and Pawlowsky [16] and Damodaran [17], a broad range of proteins induces emulsifying activity due to the presence of non-polar groups known as “surface-active” groups. As also indicated by Dickinson [18], the emulsifying capability of polysaccharides is most probably based on a small fraction of surface-active proteins attached to the gum. Therefore, the purification process maintaining the protein fraction is recommended, if the interfacial activity or emulsifying property of the gum is targeted. The present study revealed that the purification Method C led to maintain the highest protein content among all purification techniques. Therefore, the purification using saturated barium hydroxide is recommended, if the interfacial activity or emulsifying property of heteropolysaccharide-protein biopolymer from durian seed is targeted. On the other hand, the purification process using Fehling solution is recommended, if the biological aspect of the gum is targeted.

If the polysaccharide-protein structure is highly soluble in water, forming “compact globular” structures, such a globular structure will provide the capacity to reduce the surface tension of aqueous solutions [19,20]. As reported by previous researchers [21,22], this interfacial activity of gum Arabic could be due to the presence of small amounts of the protein fraction as an integral part of the gum structure. In the current study, the protein fraction present in molecular structure of the heteropolysaccharide-protein biopolymer might be responsible for the interfacial activity of the biopolymer in the emulsion system. In addition, the chemical structure and content of protein fraction significantly influence the solubility of the heteropolysaccharide-protein biopolymer from durian seed.

The current study revealed that none of purification techniques totally eliminated the protein attached to the gum structure. This observation was also reported by previous researchers [13,23]. Westphal and Jann [24] also reported that the partition coefficient in biphasic phenol–water mixtures allowed the complete extraction of the protein fraction from aqueous polysaccharide solutions in a one-step operation under controlled conditions (*i.e.*, pH and ionic strength). As stated by Westphal and Jann [24], the mixture of phenol and water has a high dielectric constant; where protein is soluble in phenol and polysaccharide is soluble in water. Bouzouita *et al.* [6] reported that the purification process led to reduced ash and protein contents. Cunha *et al.* [15] also found that four different purification methods would reduce the protein content of crude guar gum (3.64%). They reported different protein contents (0.00–1.06) in purified guar gum depending on the purification process. Youseff *et al.* [13] also illustrated that the phenol-solvent purification of fenugreek gum reduced the protein content to 0.16%. Garti *et al.* [25] reported high protein reduction (0.80%–0.95%) after using a physical separation method. Previous researchers [14] reported that an enzymatic treatment of fenugreek gum led to a decrease in the protein content to 0.6%. López-Franco *et al.* [23] observed that the protein content of mesquite gum was not significantly decreased by the purification processes. They concluded that the gum did not contain "free low molecular weight peptide”. 

The protein content of purified heteropolysaccharide-protein biopolymer from durian seed was higher than that of reported for purified fenugreek gum (2.36%) [14] and purified locust bean gum (0.61%–2.46%) [6]. Previous researchers [26] also reported different protein contents for various commercial gums such as fenugreek gum (13.9%), flaxseed gum (14.9%), locust bean gum (6.4%), yellow mustard gum (5.3%), pectin (7.9%), gum Arabic (1.8%) and guar (4.2%). As shown in Figure 2, the purification methods D and C resulted in significantly (*p* < 0.05) higher and lower reductions of protein content among all purification methods. The high efficiency of Fehling solution to reduce the protein fraction was also reported by Cunha *et al.* [15], and could be related to the reagent utilized. As mentioned by previous researchers [15], the complexation of the copper ions from the Fehling solution with protein may precipitate free proteins. Consequently, O-linked proteins may also be removed, due to the high concentration of the NaOH solution present in the Fehling solution, this part being cleaved in NaOH solutions with a concentration higher than 0.1 M [15].

### 2.3. Solubility

Hydrocolloids entrap a large amount of water between the chains and branches present in their molecular structures. Full solubility is beneficial from the view point of appearance and texture. Therefore, it is necessary to achieve the maximum solubilization in order to maintain the functionality. On the other hand, the crude gum does not show high solubility due to the presence of impurities and insoluble matters. The perfect elimination of these undesirable impurities depends on the purification conditions. For example, the chemical purification process is more efficient to reduce the content of impurities rather than the simple solubilization of the gum and separation of the insoluble matters only by simple centrifugation [27]. Hydrocolloids behave differently from common food ingredients due to their hydration process [28]. When ordinary food ingredients such as sucrose are exposed to water, the particle size decreases, and the substance is fully dissolved in water, while hydrocolloids behave differently in water. When a hydrocolloid is added to water, it swells like a sponge by absorbing water. In most cases, the particle size increases, and the hydrocolloid is not fully dissolved in water [29].

**Figure 3:**
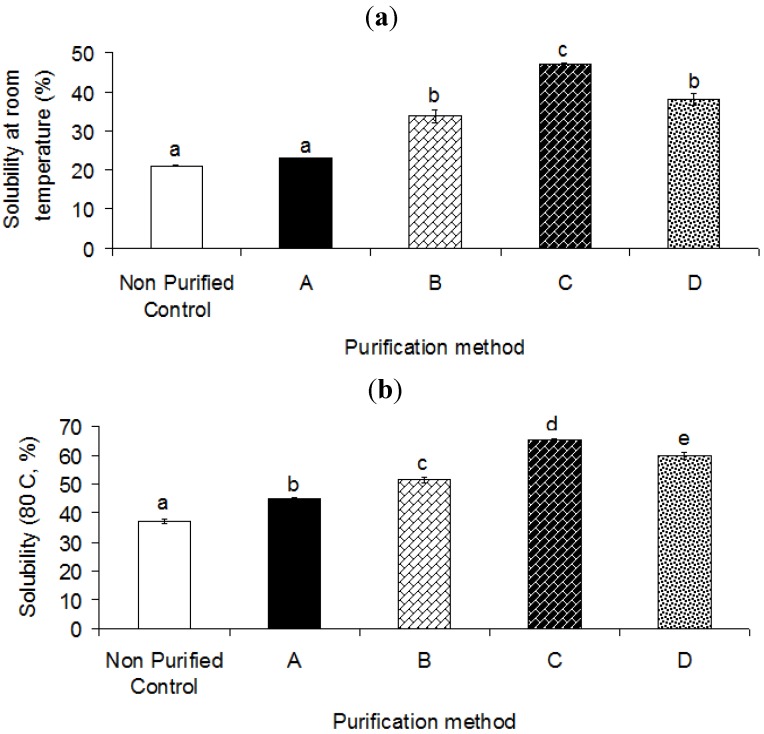
Effect of different purification methods on the solubility of hetero-polysaccharide-protein biopolymer from durian seed at (**a**) room temperature; (**b**) elevated temperature (80 °C). A, Isopropanol and ethanol; B, Isopropanol and acetone; C, Saturated barium hydroxide; D, Fehling solution.

In the current study, the chemical purification along with further centrifugation was considered to provide a more efficient process for reducing the content of impurities. The solubility of crude and purified heteropolysaccharide-protein biopolymer from durian seed is shown in Figure 3.

As obvious in Figure 3, the purification process significantly (*p* < 0.05) improved the solubility of crude durian seed gum, which varied from 23.1% to 46.8% as compared to the control sample (21.4%) (Figure 3a). The efficiency of the purification process was mainly dependent upon the purification procedure and composition of the utilized solvents and reagents. The solubility of natural gums depends on the ratio of soluble to insoluble matters. Therefore, the increase in solubility could be related to a reduction of the insoluble matter during the purification process.

The water solubility of a homogenous polysaccharide is different from that of a polysaccharide-protein structure (or heterogeneous polysaccharide). As stated by previous researchers [30], the interactions between protein and polysaccharides may be compatible or incompatible in the aqueous solution from a thermodynamic point of view. According to Tolstoguzov [31], protein-polysaccharide structures may lead to three different interaction patterns: (i) compatible or co-solubility; (ii) association or complex coacervation and (iii) incompatibility. The compatible or co-solubility is restricted to highly diluted solutions. In association, the phase separation of both biopolymers from the phase containing solvent takes place during the complex coacervation [30]. However, the most usual pattern corresponds to thermodynamic incompatibility between protein and polysaccharide molecules, thus reducing the solubility of protein-polysaccharide polymer in the aqueous solution. This incompatibility results in the formation of two separate phases, each being rich in one of the biopolymers [16].

The crude gum showed a relatively low solubility, even at high temperature. The presence of impurities, high molecular weight or large molecules and insoluble matters, which significantly affect the solubility of gum, can be responsible for the low solubility of the crude gum. In fact, the changes in solubility could be explained by the significant effect of the purification process on the main chemical composition (e.g., carbohydrate composition and protein fractions) and impurities of the crude durian seed gum. The increase in solubility could be interpreted by the fact that the purification process led to a reduction in the content of hydrophobic compounds (e.g., fat) and insoluble impurities (e.g., husk, fiber, ash, natural pigments and enzymes). In the current work, the increase in solubility could also be due to the reduction of protein content during the purification process. The purification also leads to reduced ash and protein contents, thereby enhancing the solubility [6,27]. As also stated by Lopez da Silva and Gonçalves [27], the presence of proteins, ash and enzymes can diminish the solubility of the crude gum. They reported that fat, fiber, ash and protein contents of locust bean gum were drastically reduced after the purification process.

The cellulose fraction is considered as one of the main impurities which negatively affect the solubility of crude gum. However, it is mostly removed by the purification process, depending on the efficiency of the purification method [32]. The low solubility could also be explained by the presence of a greater number of small molecules in purified gum C rather than in purified gum A. The low solubility of the purified gum A may also be attributed to the possible changes in the molecular structure of gum induced by the extraction using hot water (80 °C) for 6 h, which causes the gum to be less soluble at ambient temperature. Lopez da Silva and Gonçalves [27] showed that the purified locust bean gum was almost totally soluble (~98% against 89% for the crude gum), thus providing clearer and more stable solutions than the crude gum. They also found that the small and highly substituted molecules with high galactose content showed more solubility than the purified gum with low galactose content [27]. Doyle *et al.* [33] also reported that the solubility of Ivory nut mannan from *Phytelephas macrocarpa* increased with increasing the content of galactose; while Ivory nut mannan containing almost no galactose side chains was insoluble in water. 

Although, the crude gum contained high galactose content, it showed the least solubility as compared to the purified seed gums. The results indicated that the solubility of the crude gum was influenced by not only the galactose content, but also the type and content of impurities as well as the size and weight of molecules. In fact, the low solubility of crude gum could be related to the presence of impurities, high molecular weight or large molecules and insoluble matters which significantly affected the solubility of gum. Although the purified gum B showed the smallest particle size, the purified gum C exhibited the highest solubility. This indicated that the presence of small particles may actually reduce the degree of solubility. When the particle size is too small, some cellulose may be suspended during the extraction process and remained in the supernatant liquid. The extracted cellulose fraction is counted as part of the gum impurities, though it is mostly removed by the purification process [32]. Previous researchers [34] reported that the degree of galactose substitution affects water solubility. They showed that the molecular size and the refined structure (M/G ratio, galactose distribution in the mannose linear chain) influenced the solubility mechanism. Brummer *et al.* [14] compared different gums with similar molecular weight, but different M/G ratio. They found that the gum containing low galactose tends to have higher intrinsic viscosity, thus negatively affecting the solubility of the gums. 

Showing the same trend as the solubility at ambient temperature, the purification process significantly (*p* < 0.05) improved the solubility of the crude gum at an elevated temperature (80 °C). As shown in Figure 3b, the purified durian seed gums showed different levels of solubility ranging from 29% to 52% as compared to the solubility of the control sample (19%). In general, the purified seed gums showed higher solubility than the crude durian seed gum. Koocheki *et al.* [35] showed that the low solubility of crude *Lepidium perfoliatum* seed gum could be due to the presence of impurities in the gum. The heat-dependency solubility varies depending on the gum, its molecular structure and function. For example, a viscosity-enhancing agent requires different temperatures to reach the maximum solubility as compared to a gelling agent. Previous researchers [36,37] reported that guar gum showed high solubility in cold water; whereas locust bean gum had low solubility at ambient temperature, and heat was required to reach the maximum solubilization. 

The current study revealed that the solubility of the purified gum increased significantly (*p* < 0.05) with increasing temperature (Figure 3). In fact, the purified gum was more soluble at the elevated temperature rather than at ambient temperature. This could be explained by the fact that some molecules (*i.e.*, high molecular weight molecules and galactomannan with low galactose content) are dissolved at high temperature; while they are not soluble at low temperature [38]. Another reason is that the hydrogen (H) bonds among polysaccharide chains are broken at high temperature, and the OH-groups are exposed to the water, thus enhancing the solubility [39]. The elevated temperature may alter the soluble mass of galactose in high molecular weight polysaccharide present in the aqueous solution. In the current study, the purified gums C and A had the highest and lowest degree of solubility among all purified samples (Figure 3b). The low level of galactose substitution may result in the compact packing of polysaccharide structure in aqueous solution, thus reducing the water solubility. This was reported by previous researchers [39]. A relatively low solubility of heteropolysaccharide-protein biopolymer from durian seed could be also explained by the nature of the gum. In fact, the solubility of natural hydrocolloid is less than those containing uronic acids, which are often in the polyelectrolyte form (charged or ionic). When hydrocolloids are dissolved in water, they induce different conformation structures (spherical, random coil or rod-like). The conformation structures depend on the nature of monosaccharide and inter-sugar linkage (α or β) as well as intra- and intermolecular interactions between polysaccharide and water molecules. The interactions will determine the rheological flow behaviour of solution.

### 2.4. Water- and Oil-Holding Capacity (WHC and OHC)

Hydration properties are influenced by the swelling capacity, solubility, and water-holding capacity (WHC) [40]. Water-holding capacity (WHC) is the ability of the gum to hold water. The wide industrial application of gum exudates is due to their high water-holding capacity (WHC) to produce gels or highly viscous solutions. They can be used to reduce the vaporization rate and alter the freezing rate. They are also used to improve the texture by modifying the formation of ice crystals and water retention. It is equal to the moisture content of the gum after the equilibrium has been established under a given condition [41]. It is necessary to evaluate the WHC of gum for the viewpoint of storage conditions. In addition, it is necessary to determine the water sorption properties of the ingredient in the view point of economic potential application in any food, cosmetic and pharmaceutical product [42,43].

**Figure 4:**
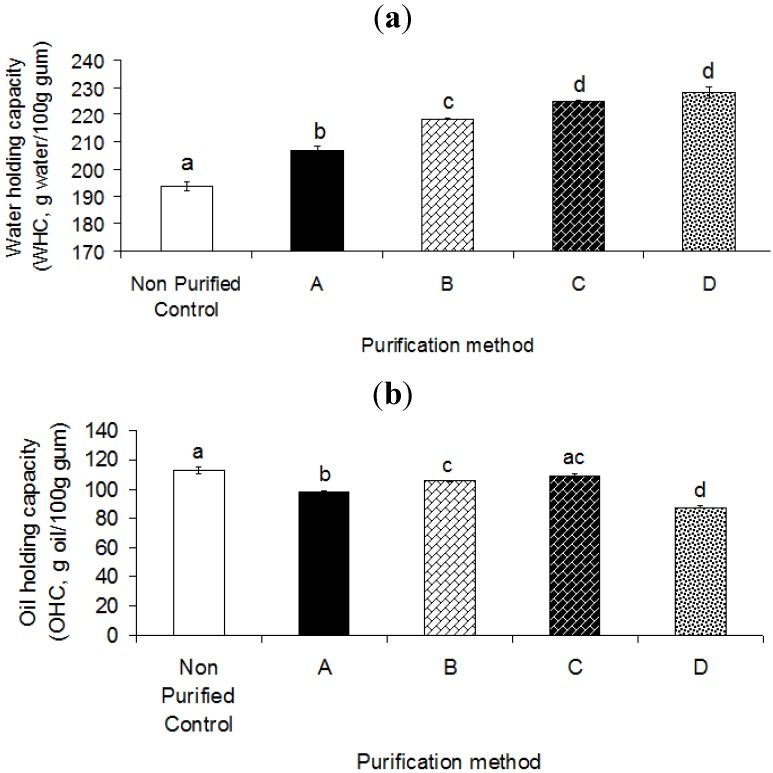
(**a**) Effect of different purification methods on the water-holding capacity (WHC). (**b**) oil-holding capacity (OHC) of heteropolysaccharide-protein biopolymer from durian seed. A, isopropanol and ethanol; B, isopropanol and acetone; C, saturated barium hydroxide; D, Fehling solution.

The results indicated that the purification process significantly (*p* < 0.05) increased the water-holding capacity (WHC) of heteropolysaccharide-protein biopolymer from durian seed (Figure 4a). The purified gums (A-D) showed high WHC ranging from ~207 to 228 (g water/100 g gum) which was higher than WHC of the control sample (~193 g water/100 g gum). This observation indicated that the purification process significantly (*p* < 0.05) improved the ability of gum to absorb water (Figure 4a). Water-holding capacity (WHC) is dependent on many factors such as extraction and purification conditions, which affect the contact area between the surface of the hydrocolloid and water. It also depends on the interaction intensity of protein with water molecules with polysaccharide molecules, hydration positions, protein configuration and environmental conditions [44]. The intensity of hydrophilic groups (such as hydroxyl groups) could also influence the extent of hydrodynamic interactions between the polysaccharide and water molecules [44].

In the current study, the purified gum D and A exhibited the highest and least WHC among all purified gums (Figure 4a). The low value of WHC of the purified gum A could be attributed to the presence of higher content of impurities present in the purified gum A. This may result in strong interactions between polysaccharide molecules and impurities, thus reducing the interaction intensity with water molecules. The ability of purified gum D and C to retain higher amounts of water was mainly attributed to the purification procedure and conditions. 

As explained by Sciarini *et al.* [39], the gum purification can cause the unfolding of the constituent polysaccharides, thus resulting in a higher proportion of ramification which affects the water absorption capacity. Hence, it is easier for water to interact with polysaccharide chains after the gum purification [39]. Iwe *et al.* [45] reported that gums extracted from the stem and root of cissus had high WHC (1,000 g water/100g gum), resulting in a viscous-jelly. The side chain and central hydrophobic stem of the polysaccharide could interact differently with the water molecules depending on the chemical composition of the gum which affected the water absorption capacity of gum [45]. The degree of WHC also depends on the particle size and distribution. A fine uniform structure with lots of small pores would probably result in higher WHC and better water retention than a coarse structure with large pores. In the current study, the purified gum D may have a finer uniform gum structure than the purified gum A, thus resulting in higher capacity of water absorption. 

Oil-holding capacity (OHC) is one the most notable functional properties of a hydrocolloid, which represents the capacity of oil absorption [46]. Some polysaccharides confer some fat characteristics by binding great quantities of fat, thereby inducing plasticity, lubricity, and melting sensation [47]. In the current study, the purification processes led to a significant (*p* < 0.05) decrease in the oil absorption capacity (Figure 4b). In fact, the purified gums showed relatively low OHC values ranging from ~82 to 110 (g oil/100 g gum) as compared to the control sample (~115 g oil/100 g gum) (Figure 4b). In fact, the control sample (or crude sample) showed higher OHC than the purified gums. This might be attributed to the presence of non-polar side chains and hydrophobic fraction (*i.e.*, fat and protein), which may bind the hydrocarbon units of oil, thus inducing a higher capacity of oil absorption [48]; while the purification process resulted in the reduction of those hydrophobic impurities, thereby reducing the OHC. A polysaccharide alone does not play a fat substitute role since its long chains prevent the formation of spherical particles, which are known as the characteristics of fat. 

The purified gum C had the highest OHC (110 g oil/100 g gum) among all purified gums (82–102 g oil/100 g gum). Conversely, the purified gum D showed the lowest OHC (82 g oil/100 g gum) (Figure 4b). The purified gum C showed almost the same OHC as the crude sample; while the other purified gums (A, B and D) exhibited significantly lower OHC than the control sample (Figure 4b). This highlighted the effectiveness of the purification methods (A, B and D) to reduce the hydrophobic (or oil soluble) fractions. In the current study, OHC levels were similar to those reported by previous researchers [45] for cissus stem gum and sweet cassava starch–cissus stem gum mixture (55–120 g oil/100 g gum). They reported that cissus stem gum showed an extremely low oil absorption capacity; therefore, it can be suitable for applications in oil-in-water emulsion systems. Galla and Dubasi [49] showed that whole-seed meal absorbed less oil as compared to dehulled–defatted gum of karaya (*Sterculia urens*). They found the reverse trend for WHC. They mentioned that the low amount of oil-holding by whole-seed meal could be due to the presence of fixed oil in the sample. This might prevent further absorption of oil. Thanatcha and Pranee [41] reported that the mucilage from *Ziziphus mauritiana* Lam had a high oil absorption capacity because of the presence of many nonpolar molecules in the crude mucilage that resulted in the entrapment of the high amounts of oil particles. 

## 3. Experimental

### 3.1. Materials and Methods

Isopropanol, ethanol (95%), absolute ethanol (99.9%), acetone, hydrochloric acid, saturated barium hydroxide, acetic acid were purchased from Fisher Scientific (Pittsburgh, PA, USA). Sunflower oil was purchased from the supermarket (Selangor, Malaysia). Durian (*D. zibethinus*) fruit was purchased from the local market (Selongor, Malaysia). Ripened durian fruits were selected based on the size uniformity and free of visual defects. The fruits were then de-husked (cut open the rind), by cutting along the suture on the back of the lobules. Durian seeds were removed, cleaned and rinsed thoroughly with sterile distilled water. There is a possibility to produce “hard seed” if the moisture is reduced. Hard durian seeds with reduced moisture content will resist germination under favorable conditions, thus prolonging the storage life. The seed was partially dried by the air circulation. The dried seeds were then packed in plastic bags and stored in a dry and cool place (10 ± 2 °C) until the extraction process [50]. All the experiments were performed with deionized water.

### 3.2. Chemical Extraction

The chemical extraction was performed according to the method described by previous researchers [51]. The successive steps of the defatting, decolouring, solvent soaking, gum dissolution, centrifugation and precipitation, were considered for the chemical extraction. Durian seed were washed and chopped into small pieces. Then, it was air dried by using the air circulation before milling into flour. The cold extraction was used to extract the oil from the durian seed flour in order to avoid the thermal degradation. The defatting process was carried out successively using hexane and isopropanol (60:40) at the room temperature (25 ± 1 °C). Our preliminary study showed that the solvent mixture containing hexane and isopropanol (60:40) was the most efficient solvent for defatting process among all studied solvents (*i.e.*, petroleum ether, hexane, isopropanol and ethanol).

The solvent residue was removed by centrifugation at 1,400 g for 15 min (Avanti J-25 Centrifuge, Beckman Coulter GmbH, Krefeld, Germany). Then, defatted-durian seed flour (1 kg) was exhaustively decolored using ethanol for a decoloring time of 120 min. The decolorized seed flour was vacuum filtered and then soaked in 1% aqueous acetic acid for 1.5 h at ambient temperature (25 ± 1 °C). Then, the slurry was filtered with nylon cloth filter and the filtrate was precipitated with 95% ethanol. The precipitated slurry was washed three times using absolute ethanol (99.9%) to achieve a very light brown amorphous crude gum. The crude gum was collected and oven dried at 40 °C for 12 h [51]. By using the crude seed gum as a control sample, the effectiveness of four different purification techniques will be determined.

### 3.3. Purification Process

#### 3.3.1. Method A

In the purification Method A, the crude seed gum was purified by using hot water, ethanol and isopropanol as described by Youssef *et al.* [13]. Initially, the gum solution (2.5% w/v) was prepared by dissolving the crude gum (25 g) in deionized water (1 L) at 80 °C water bath for 6 h, followed by stirring at room temperature overnight. The gum solution (2.5% w/v) was subjected to the centrifugation (Avanti J-25 Centrifuge, Beckman Coulter GmbH, Krefeld, Germany) for 15 min at 15,180 g. The supernatant was precipitated by the addition of absolute ethanol (1.2 L), and the supernatant was decanted. The residue was recovered and kept overnight in 100% isopropanol [52]. Finally, the residue was dried in the oven 40 °C for overnight to prepare the purified gum. 

#### 3.3.2. Method B

In the purification Method B, the purification process was carried out by using isopropanol and acetone as reported by previous researchers [6]. One g of the crude seed gum was precipitated by soaking into two volume excess of isopropanol, and allowing the gum-solvent slurry to stand for 30 min. The white fibrous precipitate was collected by the filtration using the screen (53 μm). Then, the collected precipitate was washed twice with isopropanol and acetone [52]. Finally, it was dried in the oven overnight at 40 °C.

#### 3.3.3. Method C

In the purification Method C, the crude seed gum was purified through barium complexing according to the method described by previous researchers [34]. In this method, the gum solution (2.5% w/v) was prepared by dissolving the crude gum (2.5 g) in water (100 mL) and continuouly stirring for 12 h at 60 °C. Subsequently, the gum solution was precipitated with saturated barium hydroxide solution, and the precipitate was separated by a Beckman centrifuge (Avanti J-25 Centrifuge, Fullerton, CA, USA) at 15,180 g for 15 min. The precipitate was stirred with 1 M acetic acid for 8 h and recentrifuged at 15,180 g for 15 min. Finally, the supernatant was precipitated and washed with 90% and 95% ethanol, respectively [52]. The washed precipitate was dried at oven 40 °C overnight.

#### 3.3.4. Method D

In the purification Method D, the purification was performed by using Fehling solution as reported by Cunha *et al.* [15] with some modification. Initially, the crude gum (1 g) was dissolved in water (approximately 100 mL) and stirred for 24 h with magnetic stirring. The prepared gum solution (1% w/v) was precipitated by adding freshly prepared Fehling solution (5 mL). Fehling’s solution consists of copper sulfate (34.66 g) dissolved in distilled water (500 mL). The precipitate caused by Fehling solution was collected on a glass filter (No. 3). Then, the precipitate was dissolved in 0.1 M hydrochloric acid by a magnetic stirrer for 1 h until the full solubilization. The solution was precipitated with three volumes of 95% ethanol. The precipitate was separated by the glass filter (No. 3) and washed with 95% ethanol until pH 6 [52]. Finally, the filtrate was washed with acetone and dried in the oven at 40 °C overnight.

### 3.4. Analytical Tests

#### 3.4.1. Purification Yield

The purification yield was calculated as the dry weight of the purified gum relative to the initial crude gum based on the following equation:


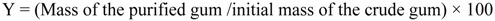
(1)

#### 3.4.2. Protein Analysis

Protein content was determined according to the method described by Nakauma *et al.* [53]. The experiment was carried out by using a VAPODEST (VAPODEST 20, rapid distillation system, Gerhardt GmbH & Co., Brackley, Northants, UK). Seed gum (approximately 1 g) and concentrated sulphuric acid (12–15 mL) were placed into a flask. Then, potassium sulphate (7 g) and a copper catalyst were added to the mixture, which was then heated up to 40 °C for 60 min. Then, hot deionized water (50 mL) was added to the mixture after cooling to 20 °C. Subsequently, 10 mL of 45% (w/v) sodium hydroxide (NaOH) solution was mixed to change the ammonium ions to ammonia gases in the higher pH. The ammonia gas was trapped by 1 M boric acid solution. Then, it was titrated with 5 mM sulphuric acid for the neutralization using methyl red and methylene blue as indicators. The protein analysis was performed in triplicate for each sample and reported based on dry basis. Protein content was calculated based on the following equation [53]:


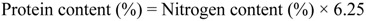
(2)

where 1 mL of sulphuric acid 5 mM = 0.14007 mg nitrogen

#### 3.4.3. Solubility

The solubility was determined according to method described by Dakia *et al.* [34], with minor modifications. One g of seed gum powder was added to distilled water (100 mL) and the mixture was stirred for 30 min. The solubility was measured by stirring the mixture at different room temperature (25 ± 1 °C) and elevated temperature (80 °C) in order to determine the effect of temperature on the solubility of the gum. The gum solution was then centrifuged at 6,000 g for 30 min to remove the insoluble material, and the supernatant was transferred to disposable Petri dishes and oven dried at 105 °C for 24 h until constant weight [50]. The solubility was calculated by the weight difference and expressed in dry basis (Equation 3). The solubility measurement was carried out in triplicate and the average of three individual measurements was considered for further data analysis.


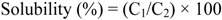
(3)

where C_1_ is the supernatant concentration (mg); C_2_ is the initial concentration (mg).

#### 3.4.4. Water- and Oil-Holding Capacity

Water-holding capacity (WHC) for of the crude and purified gums was determined according to the method described by Galla and Dubasi [49]. One g of the gum was suspended in distilled water (10 mL), vortexed for 2 min and centrifuged with a refrigerated centrifuge 3-18K (Sartorius, Sigma 3-18, Göttingen, Germany) at 3,000 g for 30 min. The free water was decanted and the water absorbed by the samples was expressed as grams of water absorbed per 100 g of seed gum. Oil-holding capacity (OHC) was also determined by dispersing the gum (1 g) in refined sunflower oil (10 mL), vortexing for 2 min and centrifuging with a refrigerated centrifuge. It was expressed as grams of oil absorbed per 100 g of seed gum [50]. The measurements were performed in triplicate for each sample. 

### 3.5. Experimental Design and Data Analysis

A completely randomized design (CRD) was considered to investigate the effect of different purification methods on the purification yield and physicochemical properties of the crude and purified biopolymer from durian seed. Four different purification methods, namely Method A (isopropanol and ethanol), Method B (isopropanol and acetone), Method C (saturated barium hydroxide) and Method D (Fehling solution) were chosen based on the preliminary study and literature. The crude durian seed gum was considered as the control sample. The effectiveness of the purification process was evaluated by comparing the physicochemical and functional properties of the purified seed gum with the control sample. The extracted yield, protein content, solubility, WHC and OHC were considered as response variables. The data was subjected to one way analysis of variance (ANOVA) to determine the significant (*p* < 0.05) differences among the purification methods. All data analysis was carried out by using Minitab version 15 (Minitab Inc., Pine Hall Road State College, PA, USA). Fisher multiple comparison test was used to evaluate significant differences (*p* < 0.05) between the different purified gums as compared to the control sample.

## 4. Conclusions

The present work revealed that all purification processes led to reduced protein content, thus enhancing the purity of the crude gum. However, none of purification techniques completely eliminated the protein fraction present in the chemical structure of the biopolymer from durian seed. Therefore, it was hypothesized that the protein fraction might be a part of the molecular structure of the durian seed biopolymer. In parallel, all purification processes significantly (*p* < 0.05) enhanced the solubility and WHC of the biopolymer from durian seed. Conversely, all purification process decreased the capacity of oil absorption (OHC) of the biopolymer. This might indicate that the chemical purification had more significant effect on the hydrophobic fraction rather than the hydrophilic fraction present in the chemical structure of the biopolymer from durian seed. In fact, the removal of insoluble matters (e.g., insoluble fiber and cellulose), protein and hydrophobic fractions from the biopolymer structure might be responsible for enhancing the solubility and WHC and reducing OHC of the purified biopolymer as compared to its crude form. Based on the purification ratio and use of chemical, Method B (isopropanol and acetone) and Method C (saturated barium hydroxide) appeared to be more economic methods than Method A (isopropanol and ethanol) and Method D (Fehling solution) for purifying the crude biopolymer from durian seed. On the other hand, Method C and Method D conferred the purified gum with more appropriate functional properties than Method A and Method B. If the physicochemical and functional properties of the heteropolysaccharide-protein biopolymer are targeted, the precipitation using saturated barium hydroxide (Method C) is recommended. If the biological application of the gum is targeted, the precipitation using Fehling solution (Method D) resulting in a more pure gum with the least protein content is recommended.
